# Mid-term prognosis of the stromal vascular fraction for knee osteoarthritis: a minimum 5-year follow-up study

**DOI:** 10.1186/s13287-022-02788-1

**Published:** 2022-03-12

**Authors:** Shengyang Zhang, Huihui Xu, Bangjian He, Mengqiang Fan, Miaomiao Xiao, Jingjing Zhang, Di Chen, Peijian Tong, Qiang Mao

**Affiliations:** 1 Department of Orthopedics, The First Affiliated Hospital of Zhejiang Chinese Medical University, Hangzhou, China; 2 The First College of Clinical Medicine, Zhejiang Chinese Medical University, Hangzhou, China; 3 Institute of Orthopedics and Traumatology, The First Affiliated Hospital of Zhejiang Chinese Medical University, Hangzhou, China; 4 Shenzhen Institutes of Advanced Technology, Chinese Academy of Sciences, Shenzhen University of Technology, Shenzhen, China; 5 Department of Orthopedics and Traumatology, Shaoxing Hospital of Traditional Chinese Medicine, Shaoxing, China

**Keywords:** Knee osteoarthritis, Stromal vascular fraction, Bone marrow lesion, Full-thickness cartilage defect, Mid-term follow-up, Prognosis

## Abstract

**Background:**

The short-term safety and efficacy of stromal vascular fraction (SVF) in treating knee osteoarthritis (KOA) have been extensively studied but the mid-term and long-term prognoses remain unknown.

**Methods:**

126 KOA patients were recruited and randomly assigned to SVF group and hyaluronic acid (HA) group (control group). The scores of visual analogue scale (VAS) and the Western Ontario and McMaster University Osteoarthritis Index (WOMAC) were assessed and compared between the two groups 1, 2, 3, and 5 years after treatment. The endpoint was defined as surgeries related to KOA or clinical scores exceeding the patient acceptable symptom state (PASS).

**Results:**

The VAS and WOMAC scores in the SVF group were significantly better than those in the HA group during the 5-year follow-up after treatment. The average responsive time to SVF treatment (61.52 months) was significantly longer than HA treatment (30.37 months). The adjusted Cox proportional hazards model showed that bone marrow lesion (BML) severity, body mass index (BMI) and treatment were independent risk factors and that the use of SVF reduced the risk of clinical failure by 2.602 times. The cartilage volume was reduced in both the SVF and control groups at 5 years but reduced less in the SVF group.

**Conclusions:**

Up to 5 years after SVF treatment, acceptable clinical state was present for approximately 60% of patients. BML severity and BMI were independent predictors of the prognosis.

*Trial Registry*: This study was retrospectively registered at Chinses Clinical Trial Registry with identifier ChiCTR2100052818 and was approved by ethics committee of the First Affiliated Hospital of Zhejiang Chinese Medical University, number 2013-X-063.

## Background

Knee osteoarthritis (KOA), the most common clinical degenerative disease, is characterized by cartilage destruction, subchondral bone damage, synovial inflammation and osteophyte formation and affects 10% of men and 16% of women over age 60 worldwide [1]. As opposed to medications and physical therapy used to treat early-stage KOA and total knee arthroplasty where KOA progresses to end-stage, emerging regenerative therapy has the potential to change this treatment paradigm [2]. The stromal vascular fraction (SVF) obtained by adipose tissue enzyme digestion contains adipose-derived stem cells (ADSCs) and progenitor cells with the ability to differentiate into a variety of cell types, such as chondrocytes, which can be a therapeutic option, and SVF is considered to be comparable to and sometimes even more effective than ADSCs due to the other functional advantages it provides over ADSCs, such as structural support [3, 4]. In recent years, several studies have addressed the short-term outcomes of SVF for KOA, demonstrating their analgesic effect and joint function improvement [5–7]. Nevertheless, owing to the high cost of SVF therapy and its hoped regenerative capacity, patients may not be content to achieve only a short-term improvement in symptoms, which can also be obtained with conservative treatment. Therefore, it is essential to clarify its mid-term efficacy, which is beneficial for the patient's choice of treatment.

Indications for such regenerative therapy are unclear. For the most part, it is highly recommended for patients with apparent cartilage damage on MRI, but the extent to which cartilage damage is prognostically meaningful is not known. Tiny cartilage defects and thinned thickness, with a prevalence of > 80% among patients with symptomatic KOA, do not appear to be disastrous [8]. However, a previous study has shown that full-thickness cartilage defects are an independent risk factor for total knee arthroplasty in asymptomatic KOA [9]; the outcome of such cartilage defects in SVF treatment and their impact on prognosis are of concern. Instead of being a pure cartilage disorder, more joint structure abnormalities contribute to the progression of KOA [10]. Upon reviewing the literature, it is also notable that bone marrow lesion (BML), characterized by bone marrow oedema, fibrosis, and necrosis, is tied to total knee arthroplasty failure [11, 12]. The relationship between BML and cartilage loss and pain is becoming increasingly recognized [13].

Therefore, we conducted a single centre, parallel group, assessor blinded, and randomized controlled clinical trial to determine the mid-term outcomes and clinical failure of SVF for KOA and whether a number of factors, including full-thickness cartilage damage and BML, are predictive of prognosis.

## Methods

### Study design

**Fig. 1:**
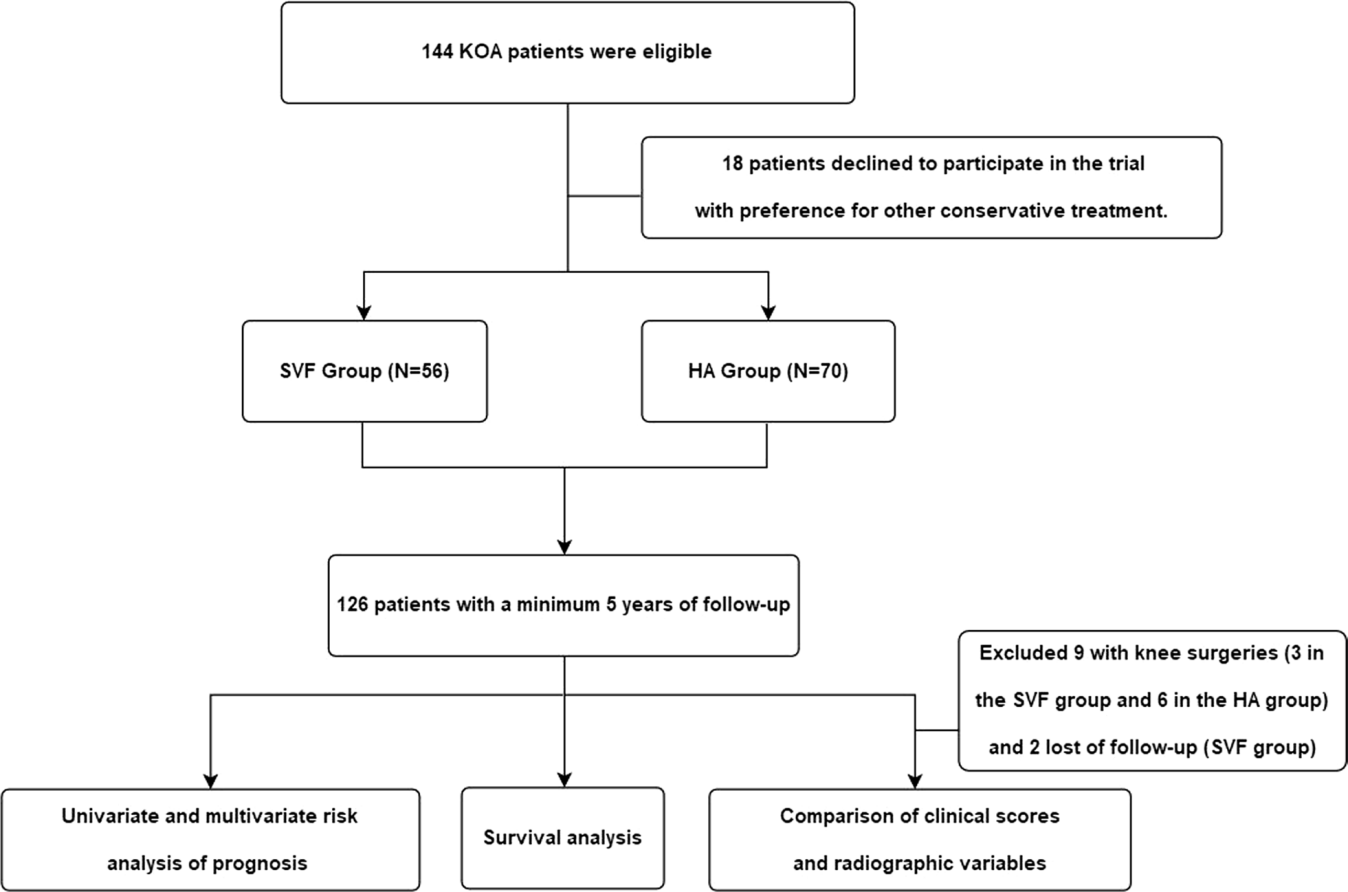
Study flow diagram

The study is a single centre, parallel group, assessor blinded, and randomized controlled clinical trial, that was retrospectively registered at Chinses Clinical Trial Registry with identifier ChiCTR2100052818 and was approved by ethics committee of the First Affiliated Hospital of Zhejiang Chinese Medical University, number 2013-X-063. KOA patients at the First Affiliated Hospital of Zhejiang Chinese Medicine University between May 2013 and July 2015 were recruited in the study (Fig. 1). The criteria included the following: the diagnosis met the diagnostic criteria in the American Rheumatism Association Revised Classification Criteria for Knee Osteoarthritis [14]; Kellgren–Lawrence (KL) grade 2–3 [15]; age 20–85 years; and no history of significant trauma. The exclusion criteria included the following: local infection of the knee joint; systemic diseases such as blood disorders or diabetes; rheumatoid arthritis, gout, autoimmune disease, or malignancy in the past 5 years; prior injection or use of oral steroids within 3 weeks before screening; knee surgery within 6 months before screening; or pain attributed to displaced meniscal tear and torn ligaments.

### Enrollment and randomization

Patients with KOA were recruited at the First Affiliated Hospital of Zhejiang Chinese Medical University. We obtained written informed consent from each eligible patient. Patients were randomly divided into the SVF group or hyaluronic acid (HA) group (control group). Randomization assignments were generated by using a computer generated, randomized number sequence, and kept the assessor who collected and analyzed outcome data blinded.

### Preparation of therapeutic SVF

Adipose tissues of patients in SVF group were obtained from the abdomen by liposuction surgery performed by a skilled orthopaedic surgeon. The patient lay supine with full exposure of the abdomen. Routine sterilization and drape operation were performed. Local anaesthesia was applied to the abdomen with 10 mg/ml lidocaine (10 ml), two small incisions of approximately 5 mm were created around the umbilicus, and approximately 40 ml of abdominal subcutaneous adipose tissue was aspirated through a sterile syringe. The incisions were closed with sutures, and the abdomen was wrapped with pressure. Harvested adipose tissue was stored in a small freezer and transported to the laboratory. The adipose tissue was washed 3–5 times with PBS containing penicillin at a 2% concentration and then centrifuged at 1000 rpm for 5 min. The upper layer of adipose tissue was removed and cut to chyme with sterilization scissors. The chylomicron adipose tissue was collected in a clean 15 ml centrifuge tube with the addition of an appropriate amount of 1% collagenase type IV at 400 rpm and 37 °C for digestion. After that, the filtrate was collected through a 100-mesh cell sieve and centrifuged at 1200 rpm for 5 min, and the supernatant was removed, the residual SVF pellet at the bottom was resuspend in PBS to a volume of 6 ml, in which 1 ml of the sample was retained for cell counting. The SVF was characterized by flow cytometry, and the constituent cell subpopulations of live nucleated SVF cells were shown as a percentage of the total number of live nucleated cells, without counting RBCs (Table 1). The remaining 5 ml, with an average count of 4.84 ± 1.61 million viable SVF cells according to the counting result, was used for injection. SVF was injected into the knee joint within one hour after successful preparation.

**Table 1:** Cell characterization by flow cytometry

Subpopulation	Avg (%)	Type pf cells
CD45−/CD31−/CD34+	33.1	SVF progenitor cells
CD45−/CD31−/CD34−	45.6	SVF non-progenitor cells
CD45−/CD31+	9.3	Endothelial cells
CD45−	92.7	Stromal vascular cells
CD45+	5.5	Leukocytes

### Intra-articular injection

The patient was placed in the supine position with the knee straight. Local sterilization was performed. 5 ml of SVF was injected into the joint cavity via a superior-lateral approach under sterile technique by percutaneous puncture with a disposable syringe once a month for a total of three times. After the injection, a local sterile dressing was applied, and the patient was instructed to bend and extend the knee joint several times. HA was injected in patients of control group as described above at a dose of 5 ml once a month for a total of three times.

### Post-injection protocol

Patients were instructed to be non-weight bearing for two days and to undertake only light activity and avoid previously painful activities for the first 3 weeks after the injection. Patients were informed of the possibility of adverse reactions, including fever, swelling, or skin rash, after the injection and were asked to contact their physician immediately if any adverse reactions occurred during the follow-up period. Patients should inform their physician to evaluate pain and function if they suffer from knee pain and take pain medication during the follow-up period.

### Primary outcomes

Pretreatment baseline data, including sex, age, body mass index (BMI), etc. were collected from both groups of patients. Patients were followed up at 1, 2, and 3 years after treatment and every 2 years thereafter, and their pain and function were evaluated by a blinded and skilled orthopaedic surgeon using the scores of visual analogue scale (VAS) (0–10 cm) and Western Ontario and McMaster University Osteoarthritis Index (WOMAC).

### Second outcomes

X-rays were used to determine the KL grade change and mechanical axis at the time of assessment, and MRI (3.0 T) was performed to evaluate cartilage structure and volume, patella-femoral pathology and BML. MRI data: a T1-weighted image, repetition time 3000 ms, echo time 33 ms, 512 × 512-pixel matrix; sagittal images were obtained at a slice thickness of 1.5 mm without an interslice gap; (2) a T2-weighted image, repetition time 4590 ms, echo time 62 ms, 320 × 320-pixel matrix; sagittal images were obtained at a slice thickness of 3 mm with an interslice gap of 3.85 mm. Cartilage structure and volume and BML were assessed over the medial tibia, medial femur, medial patella, lateral tibia, lateral femur, and lateral patella.

The KL grade and mechanical axis were assessed by a skilled orthopaedic surgeon blinded to treatment allocation and clinical data.

Patella-femoral pathology was assessed by a skilled orthopaedic surgeon blinded to treatment allocation and clinical data.

Cartilage structure and volume was assessed by a skilled orthopaedic surgeon blinded to treatment allocation and clinical data. Measurement of individual cartilage plate volumes was performed using Rhinocero 5.0 (Robert McNeel, USA) software. Contour tracing of cartilage boundaries was performed layer by layer of MRI images in isometric sections and separated from the total volume to create a cartilage 3D model [16], and the volume was calculated using the software. A full-thickness cartilage defect was defined as cartilage stripping to subchondral bone exposure, regardless of size. The full-thickness cartilage defect area of each coronal and transverse slice was measured and divided into three levels [9]: 0, no defect; 1, defect < 2 cm^2^; 2, defect ≥ 2 cm^2^.

**Fig. 2:**
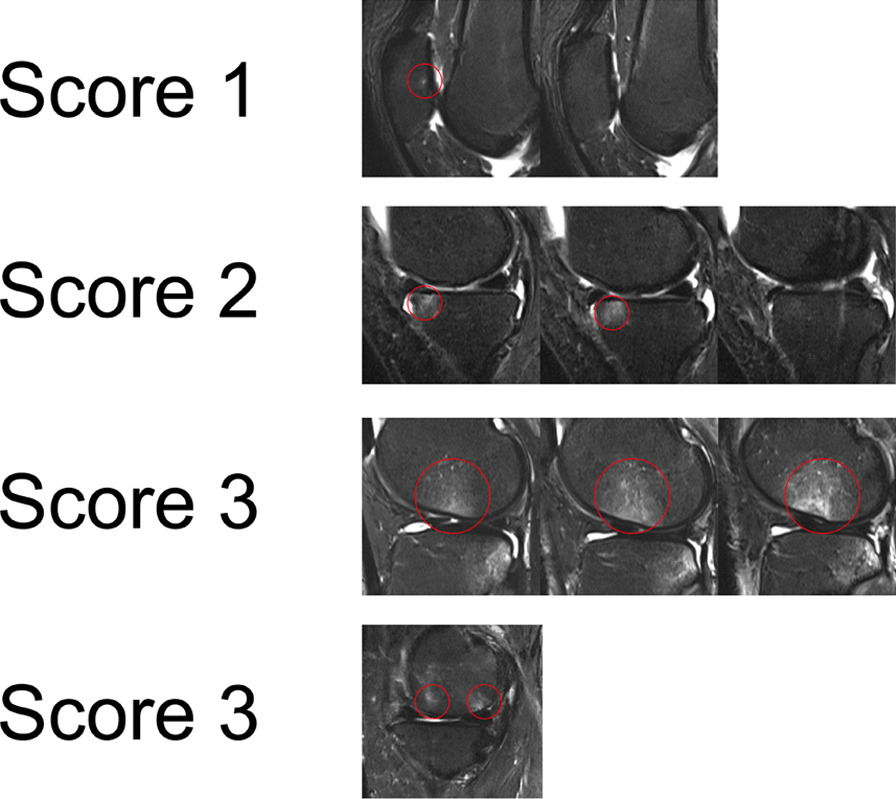
Three levels of bone marrow lesions in the red circle position. 0, no BMLs; 1, cover one slice, 2; cover two consecutive slices; 3, cover three or more consecutive slices. A score of 3 was assigned if more than one lesion was present at the same site

Subchondral BML was assessed on T2-weighted images by a skilled orthopaedic surgeon blinded to treatment allocation and clinical data and were defined as areas of high signal in the subchondral bone marrow, including cystic changes. BML size was scored by measuring the maximum area of the lesion (mm^2^) at baseline and follow-up [17]. The areas of BML in the six positions were added to determine the total size. BML severity was scored and summarized according to the number of slices covered by BML in each measurement site with reference to the previous method [18]: 0, no BML; 1, cover one slice, 2; cover two consecutive slices; 3, cover three or more consecutive slices, score 0–18. It was scored 3 if more than one lesion was present at the same site (Fig. 2).

### Definition of clinical failure

Clinical failure was defined as surgeries related to KOA, such as total knee arthroplasty, unicondylar knee arthroplasty and debridement under arthroscopy, or clinical scores exceeding the patient acceptable symptom state (PASS) (VAS > 3.23 or WOMAC function score > 31) [19]. Information about the surgery was collected at each follow-up. For patients who underwent surgery, clinical scores were not included in the final comparison analysis, but only the time of surgery was recorded for Kaplan–Meier survival analysis. We use the term responsive to denote survival for Kaplan–Meier survival analysis, which represents the knee remains responsive to treatment, i.e., the lasting impact of the treatment. To avoid overestimating responsive time, for patients with very poor clinical scores after 1 year, we carefully questioned the patient's medical history prior to that time to determine the exact time beyond PASS.

### Statistical analysis

SPSS statistics 25 software (IBM, USA) was used to perform the statistical analysis, and the data are presented as the mean ± standard deviation and percentage. Differences in baseline data were assessed by independent samples t-tests for continuous variables and chi-square tests for categorical variables. For the clinical score comparison, the main effect (within- and between-subjects) and crossover effect were analysed by two-factor repeated-measures ANOVA. The separate effects were analysed by 2-way ANOVA for grouping factors at each time point and repeated measures ANOVA for time factors. With clinical failure as the endpoint, Kaplan–Meier responsive curves were generated to compare the responsive probability of the two groups. The crude risk factors for clinical failure were obtained through univariate Cox regression using the same endpoint, and the significance level was set at *p* < 0.10. After that, with diagnosing collinearity with variance inflation factor (VIF), a multivariate Cox regression was performed to exclude confounding factors for independent risk factors and to develop independent prediction models. KL grade, mechanical axis, patella-femoral pathology, full-thickness cartilage defect, total cartilage volume and BML-related variables were mandatory to be included in the multivariate analysis. Differences were considered significant with *p* < 0.05.

## Results

### Study population

We enlisted 144 KOA patients between May 2013 and July 2015 who were accorded with inclusion criteria. 18 patients declined to participate in the trial with preference for alternative treatment. 126 patients were enrolled and randomly assigned: 56 patients in SVF group and 70 patients in the control group. Two patients in the SVF group were lost to follow-up due to a change in contact details, and 9 patients underwent surgery during the follow-up period (3 in the SVF group and 6 in the HA group); these patients were included in the Kaplan–Meier responsive analysis but not in the comparison analysis of clinical scores (Fig. 1). There were no significant differences in the baseline data between the two groups of patients (*p* > 0.05, Table 2). There were no adverse reactions during postoperative follow-up in either group.

**Table 2:** Baseline data of included patients

	SVF (*N* = 56)	HA (*N* = 70)		*p* value
*Sex*				0.692
Male	14 (25%)		16 (22.9%)	
Female	42 (75%)		54 (77.1%)	
Age, years	53.98 ± 13.69		55.63 ± 12.18	0.790
BMI	23.73 ± 2.99		23.86 ± 2.55	0.447
Mechanical axis, °	Varus 1.63 ± 2.21		Varus 1.49 ± 2.12	0.715
*KL grade*				0.366
2	41 (73.2%)		46 (65.7%)	
3	15 (26.8%)		24 (34.3%)	
*Full-thickness defect*				0.069
0	40 (71.4%)		45 (64.3%)	
1	6 (10.7%)		18 (25.7%)	
2	10 (17.9%)		7 (10%)	
Total cartilage volume (mm^3^)	16,377.16 ± 2692.40		15,851.51 ± 2143.45	0.225
BML severity	3.30 ± 4.34		2.77 ± 3.42	0.455
BML size (mm^2^)	127.68 ± 193.42		108.07 ± 149.89	0.522
Baseline VAS score	4.04 ± 1.46		3.64 ± 0.98	0.088
Baseline WOMAC score	34.57 ± 22.85		29.97 ± 19.87	0.229
Patella-femoral pathology present	28 (50%)		24 (34.3%)	0.075

SVF, stromal vascular fraction; HA, hyaluronic acid; BMI, body mass index; KL, Kellgren–Lawrence; VAS, visual analogue scale; WOMAC, Western Ontario and McMaster University Osteoarthritis Index; BML, bone marrow lesion

### Primary outcomes

**Fig. 3:**
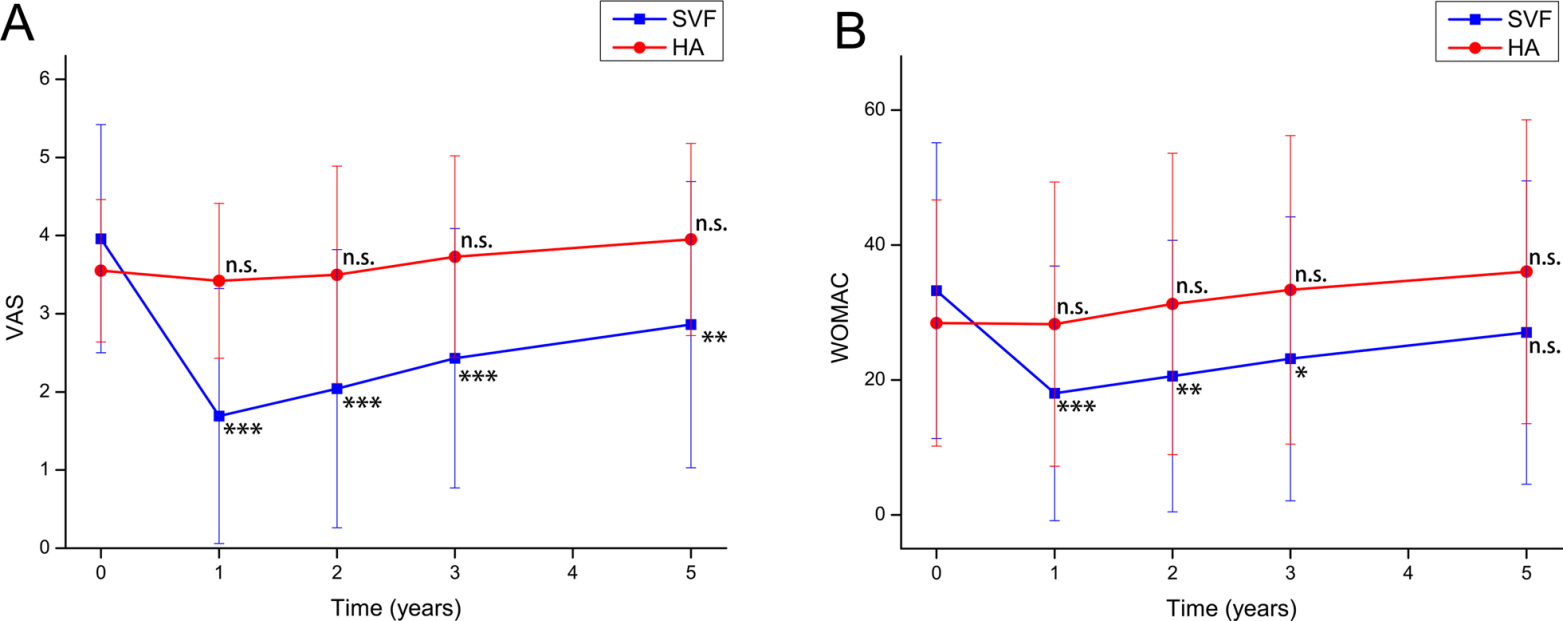
Changes in the VAS score and the WOMAC score during the 5-year period before and after treatment in the SVF group and control group. **A** The mean VAS score change. **B** The mean WOMAC total score change. *p value < 0.05, **p value < 0.01, ***p value < 0.001, ns, non-significant (*p* value > 0.05), compared with pre-treatment

A total of 115 patients at 1 year, 2 years, 3 years, and 5 years received a complete clinical score evaluation, including 51 in the SVF group and 64 in the HA group. The comparison of VAS scores and WOMAC scores between the SVF group and HA group before and after treatment is shown in Tables 3 and 4 and Fig. 3. There was a significant difference in clinical scores between time before and after treatment (VAS: *F* = 64.348, *p* < 0.001; WOMAC: *F* = 45.087, *p* < 0.001), with separate effect analyses in the SVF group (*F*[VAS] = 75.990, *F*[WOMAC] = 36.195) and HA groups (*F*[VAS] = 9.067, *F*[WOMAC] = 46.619), all at *p* < 0.001. The VAS and WOMAC scores in the SVF group were lowest after 1 year and then increased annually but remained lower than pretreatment scores at 5 years. The VAS and WOMAC scores in the HA group did not change much from pretreatment at 1 year, then increased annually and were significantly higher than pretreatment at 5 years. The post hoc tests were conducted for different time points in the HA and SVF groups. VAS scores in the SVF group were significantly lower than pre-treatment at all post-treatment time points (*p* < 0.05), and WOMAC scores in the SVF group were significantly lower than pre-treatment at years 1, 2, and 3 post-treatment (*p* < 0.05), but did not differ from pre-treatment at year 5 (*p* > 0.05). VAS scores and WOMAC scores in the HA group did not differ from pre-treatment at all post-treatment time points (*p* > 0.05). The VAS scores in the SVF group were significantly lower than those in the HA group overall after treatment (*F* = 18.030, *p* < 0.001), and the WOMAC scores were not significantly different between the two groups in the overall effect (*F* = 3.335, *p* > 0.05). Due to a crossover effect between treatment and time (*F*[VAS] = 49.319, *p* < 0.001; *F*[WOMAC] = 41.307, *p* < 0.001). We performed an analysis of the separate effects for each time point. The VAS and WOMAC scores of the SVF group were significantly lower than those of the HA group at all time points after treatment (*p* < 0.05).

**Table 3:** Comparison of the VAS scores before and after treatment in the SVF and the control group

Before or after treatment								
Group	Pre-treatment	1 year	2 years	3 years	5 years	Sum	*F*	*p* value
SVF	3.96 ± 1.46	1.69 ± 1.63***	2.04 ± 1.78***	2.43 ± 1.66***	2.86 ± 1.83**	2.60 ± 1.84	75.990	< 0.001
HA	3.55 ± 0.91	3.42 ± 0.99^ns^	3.50 ± 1.39^ns^	3.73 ± 1.29^ns^	3.95 ± 1.23^ns^	3.63 ± 1.18	9.067	< 0.001
Sum	3.73 ± 1.19	2.65 ± 1.57	2.85 ± 1.73	3.16 ± 1.60	3.47 ± 1.61	3.17 ± 1.59^a^	64.378^a^	< 0.001^a^
*F*	2.414	42.441	30.065	23.921	16.751	18.030^a^	(*F* = 49.319 *p* value < 0.001)^b^	
*p* value	0.121	< 0.001	< 0.001	< 0.001	< 0.001	< 0.001^a^		

SVF, stromal vascular fraction; HA, hyaluronic acid

^a^*F* statistic and *p* value of main effect

^b^*F* statistic and *p* value of crossover effect

^*^p value < 0.05; **p value < 0.01; ***p value < 0.001; ns, non-significant (*p* value > 0.05), compared with pre-treatment

**Table 4:** Comparison of the WOMAC total score before and after treatment in the SVF and the control group

Before or after treatment								
Group	Pre-treatment	1 year	2 years	3 years	5 years	Sum	*F*	*p* value
SVF	33.24 ± 21.93	18.02 ± 18.87***	20.57 ± 20.13**	23.14 ± 21.03*	27.04 ± 22.47^ns^	24.40 ± 21.43	36.195	< 0.001
HA	28.44 ± 18.23	28.27 ± 21.07^ns^	31.28 ± 22.33^ns^	33.36 ± 22.88^ns^	36.05 ± 22.52^ns^	31.48 ± 21.54	46.619	< 0.001
Sum	30.57 ± 20.00	23.72 ± 20.68	26.53 ± 21.95	28.83 ± 22.57	32.05 ± 22.85	28.34 ± 21.7^a^	45.087^a^	< 0.001^a^
*F*	1.449	6.609	7.015	6.378	5.108	3.335^a^	(*F* = 41.307 *p* value < 0.001)^b^	
*p* value	0.229	0.010	0.008	0.012	0.024	0.070^a^		

SVF, stromal vascular fraction; HA, hyaluronic acid

^a^*F* statistic and *p* value of the main effect

^b^*F* statistic and *p* value of the crossover effect

^*^p value < 0.05; **p value < 0.01; ***p value < 0.001; ns, non-significant (*p* value > 0.05), compared with pre-treatment

**Fig. 4:**
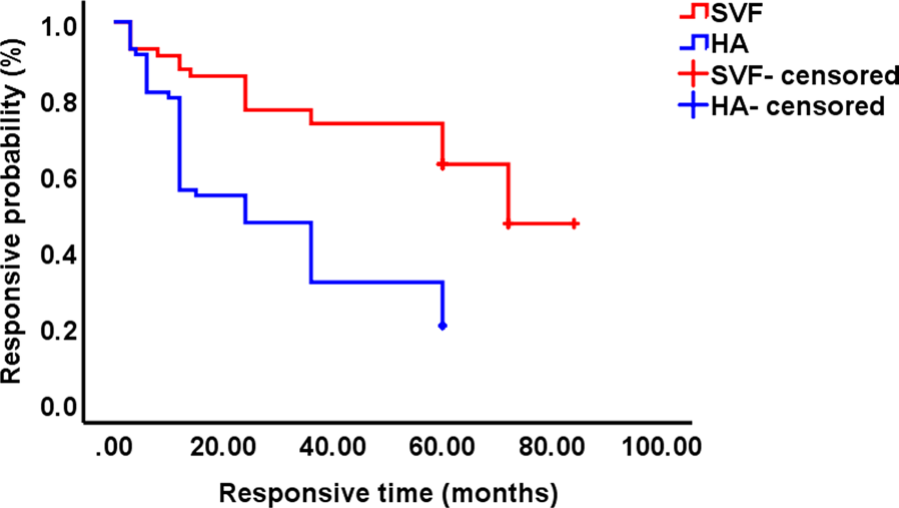
Kaplan–Meier responsive curve with clinical failure as the endpoint

To observe the clinical outcome of each patient more accurately, the Kaplan–Meier responsive curves of all patients in the two groups were plotted and compared. The SVF group showed a responsive rate of 62.5% (35/56) at the 5-year follow-up, and the rate in the HA group was 20% (14/70). According to the log-rank analysis, the mean responsive time (61.52 ± 4.14 months) of the SVF group was significantly longer than that of the HA group (30.37 ± 2.69 months) (*p* < 0.001, Fig. 4).

### Secondary outcomes

**Fig. 5:**
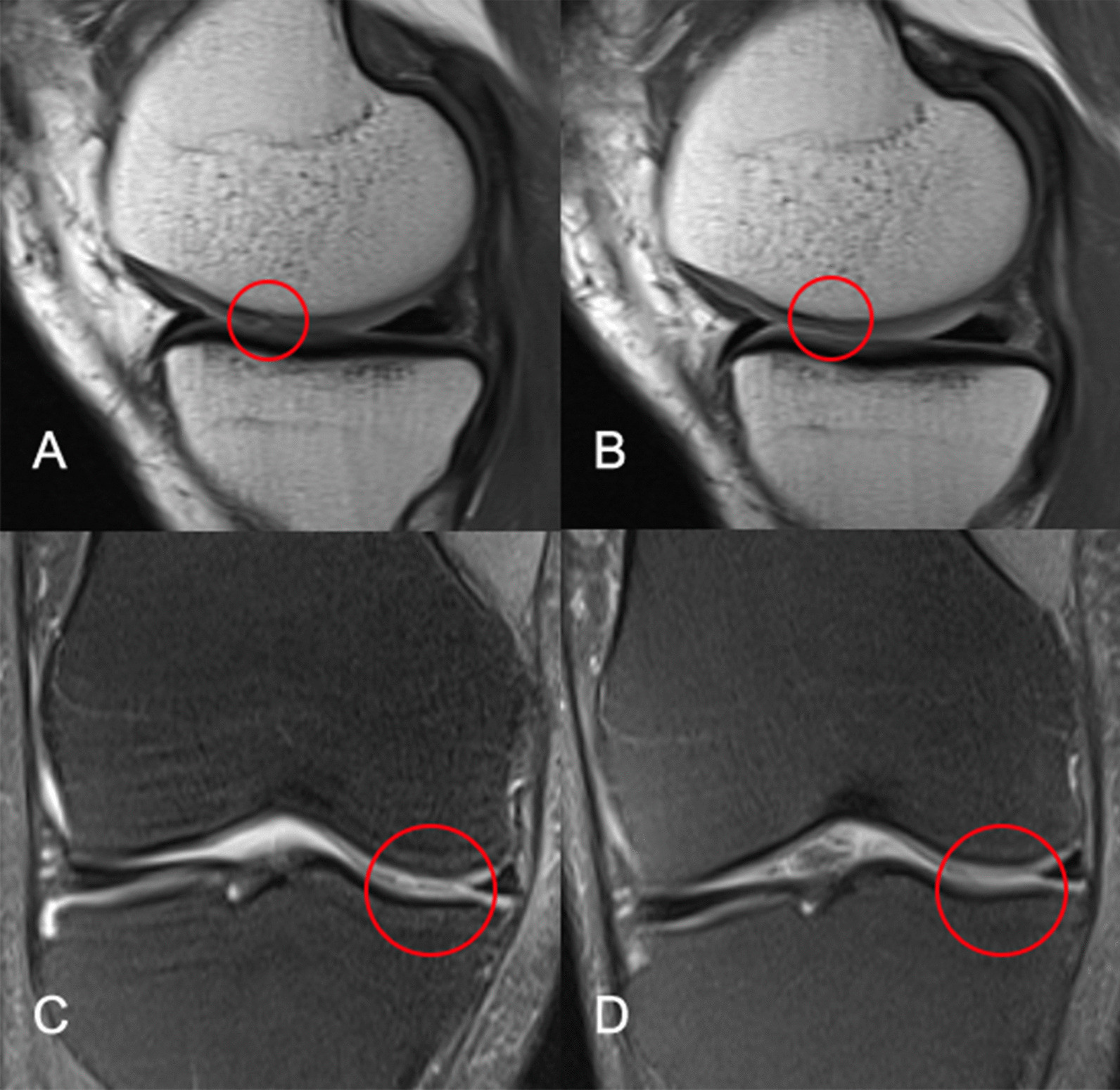
MRI evaluation of full-thickness cartilage defect changes at 5 years. **A**, **C** Coronal and sagittal images of the medial femur and tibia before injection of SVF. A grade 1 full-thickness cartilage defect can be observed in the circle. **B** Coronal and sagittal images of the medial femur and tibia 5 years after SVF injection. The full-thickness cartilage defect in the circled area disappeared, and the cartilage edge was smooth

The radiological changes as secondary outcomes at 5 years are documented in Table 5. At the final radiological examination, the total cartilage volume was significantly reduced in both groups from baseline to 5 years and was less in the HA group than in the SVF group at 5 years. Compared to the HA group, a higher percentage of patients in the SVF group had a reduced or unchanged grade of full-thickness cartilage defects, and a lower percentage of patients experienced progression (Fig. 5). There was no significant difference in BML size, severity, patella-femoral pathology or mechanical axis from baseline to 5 years and no difference between the two groups. There was no significant difference in the change in KL grade from baseline to 5 years between the two groups.

**Table 5:** Changes in radiographic variables

		SVF (*N* = 51)	HA (*N* = 64)	*p* value
BML size, mm^2^	Baseline	123.48 ± 197.02	105.49 ± 151.12	0.581
	5 years	90.33 ± 141.01	95.54 ± 146.76	0.848
	*p* value	0.149	0.516	
BML severity	Baseline	3.02 ± 4.14	2.64 ± 3.34	0.588
	5 years	2.59 ± 3.16	2.56 ± 3.30	0.966
	*p* value	0.125	0.773	
Total cartilage volume, mm^3^	Baseline	16,467.89 ± 2739.13	15,718.20 ± 2071.90	0.109
	5 years	15,121.11 ± 3174.45	13,473.30 ± 2489.59	0.003
	*p* value	< 0.001	< 0.001	
Mechanical axis, °	Baseline	Varus 1.48 ± 2.16	Varus 1.24 ± 2.02	0.536
	5 years	Varus 1.75 ± 2.11	Varus 1.40 ± 2.03	0.373
	*p* value	0.164	0.208	
Patella-femoral pathology	Baseline	25 (49.0%)	22(34.4%)	0.112
	5 years	30 (58.8%)	29 (45.3%)	0.150
	*p* value	0.321	0.206	
Full-thickness defect	Decrease	3 (5.9%)	0 (0%)	0.043
	No change	44 (86.3%)	52 (81.3%)	
	Increase	4 (7.8%)	12 (18.8%)	
KL grade	Decrease	0 (0%)	0 (0%)	0.524
	No change	43 (84.3%)	51 (79.7%)	
	Increase	8 (15.7%)	13 (20.3%)	

SVF, stromal vascular fraction; HA, hyaluronic acid; KL, Kellgren–Lawrence; BML, bone marrow lesion

### Univariate (unadjusted) risk factors

Univariate Cox regression results indicated age (per year increase, HR 1.032; 95% CI 1.013–1.051; *p* = 0.001), BMI (per point increase, HR 1.111; 95% CI 1.032–1.197; *p* = 0.005), treatment (SVF vs HA, HR 3.067; 95% CI 1.849–5.089; *p* < 0.001), KL grade (2 vs 3, HR 1.718; 95% CI 1.277–2.311; *p* < 0.001), mechanical axis (per degree increase, HR 1.101; 95% CI 0.993–1.221; *p* = 0.068), full-thickness cartilage defect (per grade increase, HR 1.581; 95% CI 1.177–2.123; *p* = 0.002), total cartilage volume (per mm^3^ increase, HR 1.000; 95% CI 1.000–1.000; *p* = 0.018), BML size (per mm^2^ increase, HR 1.002; 95% CI 1.001–1.003; *p* < 0.001) and BML severity (per point increase, HR 1.154; 95% CI 1.093–1.219; *p* < 0.001) as possible risk factors. Sex and patella-femoral pathology were not risk factors in the unadjusted analysis (*p* > 0.1). The results are shown in Table 6.

**Table 6:** Unadjusted and adjusted risk of clinical failure

Variable	Univariate analysis		Multivariate analysis	
	*p* value	Unadjusted HR (95% CI)	*p* value	Adjusted HR (95% CI)
Sex (male vs female)	0.420	1.248 (0.728–2.139)		
Age (per year increase)	0.001	1.032 (1.013–1.051)	0.140	1.014 (0.995–1.034)
BMI (per point increase)	0.005	1.111 (1.032–1.197)	0.043	1.096 (1.003–1.198)
Treatment (SVF vs HA)	< 0.001	3.067 (1.849–5.089)	< 0.001	3.602 (2.116–6.131)
KL grade (2 vs 3)	< 0.001	1.718 (1.277–2.311)	0.218	1.277 (0.865–1.885)
Mechanical axis (per degree increase)	0.068	1.101 (0.993–1.221)	0.689	1.023 (0.917–1.141)
Full-thickness cartilage defect (per grade increase)	0.002	1.581 (1.177–2.123)	0.403	1.158 (0.821–1.634)
Total cartilage volume (per mm^3^ increase)	0.018	1.000 (1.000–1.000)	0.919	1.000 (1.000–1.000)
BML size (per mm^2^ increase)	< 0.001	1.002 (1.001–1.003)	0.627	1.000 (0.999–1.002)
BML severity (per point increase)	< 0.001	1.154 (1.093–1.219)	0.024	1.104 (1.013–1.202)
Patella-femoral pathology	0.310	1.263 (0.805–1.981)	0.873	0.960 (0.578–1.592)

HR, hazard ratio; CI, confidence interval; BMI, Body mass index; KL, Kellgren–Lawrence; BML, bone marrow lesion

### Independent risk factors

Linear regression showed no collinearity (VIF < 3) for age, BMI, treatment, KL grade, mechanical axis, patella-femoral pathology, full-thickness cartilage defect, total cartilage volume, BML size or BML severity score. In the final multivariate Cox regression model, BML severity score (per point increase, HR 1.104; 95% CI 1.013–1.202; *p* = 0.024), BMI (per point increase, HR 1.096; 95% CI 1.003–1.198; *p* = 0.043) and treatment (SVF vs HA, HR 3.602; 95% CI 2.116–6.131; *p* < 0.001) were independent risk factors for prognosis. The use of SVF reduces the risk of clinical failure by 2.602 times compared with HA. Each score increase in BML severity increased the risk of clinical failure by 0.104 times. Each score increase in body mass index increased the risk of clinical failure by 0.096 times. Age, KL grade, mechanical axis, patella-femoral pathology, total cartilage volume, full-thickness cartilage defects and BML size were not risk factors in the overall model (*p* > 0.05) after adjusting for confounding factors. The results are shown in Table 6.

## Discussion

The most important finding of this study is that acceptable clinical state was present for approximately 60% of patients after SVF treatment. In terms of change in pain scores, the SVF group was superior to the control group, and although the WOMAC scores did not show an advantage for HA in overall effect, a crossover effect of time and grouping was present, and we turned to assess the scores at each time point alone, which were superior to the control group. Although the difference in WOMAC scores was not significant in the SVF group compared to pre-treatment after 5 years, it was still better than the control group However, what is not sufficiently convincing is that reports based on the mean and standard deviation of clinical score changes often reflect the average level of the subject and do not address the individual patient's perspective, making it difficult to determine the efficacy [20]. Therefore, we defined clinical failure based on the above evaluation and set it as KOA-related surgery and scores that did not meet the PASS, which fit the patients' own willingness to accept the symptoms. Comparing the responsive curves of the two groups, the 5-year responsive rate of SVF group was significantly better than that of the control group and exceeded 60%, indicating that patients treated with SVF were less likely to experience clinical failure in 5 years. Tran et al. [5] observed an improvement in clinical symptoms in SVF-treated patients after two years of follow-up, in which they attributed to paracrine mechanisms related to the anti-inflammatory effects of cell therapy. However, for mid-term prognosis, there is no evidence that the anti-inflammatory effect could be sustained over such a long period of time, so cartilage changes remain a significant consideration. Imaging of 5-years postoperative results revealed that cartilage volume was reduced in both groups compared to the preoperative period, but total cartilage volume was still higher in the SVF group than in the HA group, and a small proportion of SVF patients showed signs of repair of the full-thickness cartilage defect. This is consistent with a previous short-term study in which Song et al. observed an increase in cartilage volume in patients treated with ADSCs for 72 weeks, which began to decrease at 96 weeks [21]. This phenomenon may be related to an unavoidable natural consequence of ageing [22, 23]. Regarding the mechanism of cartilage volume effect, we suggest that firstly, SVF may promote cartilage regeneration through specific differentiation and paracrine signalling of different cell groups [24], but there is no evidence that SVF cells can directly differentiate into chondrocytes or tissues in human body. Similar to this study, several short-term clinical studies have observed the repair of cartilage defects and the widening of joint space by MRI, which indicates the result of cartilage regeneration but the processes involved need further study [5, 6, 25]. Secondly, inflammatory factors such as IL-1 and TNF-α play an important role in the progression of OA, which can promote the release of matrix metalloproteinases and make the catabolism of articular cartilage [26, 27]. After injection of SVF into the knee joint, the ADSCs produced IL-1 receptor antagonists and the tissue protective protein tumor necrosis factor-stimulated gene-6 (TSG-6), and exerted anti-inflammatory effects on chondrocytes and synovial cells via prostaglandin E2 [28, 29]. In addition, ADSCs promote the polarization of non-polarized macrophages and mature dendritic cells towards anti-inflammatory and phagocytic phenotypes [30]. Other substances in SVF may also play an anti-inflammatory role. Morris et al. [31] found that macrophages (CD11b) in adipose tissue accounted for 20% of the cells obtained from SVF, 70% of which were positive for CD301, a marker of M2 macrophages, which has anti-inflammatory and pro-angiogenic functions. And in the fat grafting procedure performed by Dong et al. [32], the inclusion of SVF resulted in increased expression of CD206 (another phenotypic marker of M2 macrophages) and negative regulation of the pro-inflammatory agents IL-1β and IL-6.The reduction in inflammation resulted in less cartilage damage, destruction, and cartilage regeneration occurred in the SVF group, but not in the HA group, ultimately causing less cartilage volume loss in the SVF group than in the HA group, although cartilage in both groups still inevitably degenerated. In addition to ADSCs, SVF contains heterogeneous cell types and different factors with paracrine effects, which may result in more significant benefits and cartilage healing potential [33]. Maintenance of existing MSCs and their functions through molecular and structural synergy is a possible mechanism. Traktuyev et al. [34] demonstrated that certain factors produced by MSCs in SVF, such as VEGF, enable better migration and survival of endothelial precursor cells (EPCs), while EPCs, by producing PDGF-BB, in turn enable MSCs to proliferate and migrate to the site of injuried tissue. Other differentiated cells such as progenitor cells in SVF may also promote cartilage regeneration. Zhao et al. measured the composition changes of articular cartilage in KOA patients before and after intra-articular injection of adipose-derived progenitor cells by multi-compositional MRI, and observed the improvement of articular cartilage [35]. We speculate that the cartilage volume advantage achieved by SVF treatment over controls may be more relevant to mid-term clinical acceptable state.

Adipose-derived cell therapies commonly use culture-expanded ADSCs and SVF. Agarwal et al. meta-analyzed 18 studies of ADSCs and SVF for KOA and concluded that although the dose or number of injections of ADSCs or SVF varied, patients showed improvement in pain and function from 2 to 24 months postoperatively [36], which is consistent with our short-term results, suggesting that compared to ADSCs, less preparation for SVF injections may be an advantage, as both therapies have achieved good clinical outcomes. The amount of ADSCs in SVF tends to be less than culture-expanded ADSCs, but the effect of ADSCs dose on efficacy is controversial. Jo et al. found more significant improvement in KOA pain at high cell doses (1 × 10^8^ cells) [37], while another study showed better results at lower doses (2 × 10^6^ cells) [38]. There are few clinical studies directly comparing the two therapies, only Yakota et al. conducted a study in this area and found that ADSCs were more clinically significant than SVF for KOA with more rapid action and fewer complications after a 6-month follow-up [39]. However, a recent animal study showed that SVF was more effective than culture-expanded ADSCs in the short-term repair of damaged cartilage and reduction of inflammatory factors such as IL-6 and TNFα in the synovial fluid [40]. Clinical studies on the differences in cartilage repair between the two therapies have not been reported. Therefore, more clinical studies are still needed to draw strong conclusions about which treatment is better. Based on the available evidence in the literature, we need to be aware that SVF is still a good treatment option.

To the best of our knowledge, our study has a mid follow-up period and exploring the factors influencing prognosis for the first time. BML severity which reflects the depth of spread of BML in bone tissue at multiple MRI slices was an independent predictor of prognosis after adjusting for confounding factors rather than cartilage-related variables. The pathology of BML is often thought to be related to bone resorption, with continued progression of BML secondary to the expansion of the area of necrosis, fibrosis subchondral tideline drift and subchondral remodelling, leading to focal, vertical shear stresses [41–43], which accelerate loss of cartilage so that BML seem to be dominant. In this study, we also focused on BML size, as the largest area shown on a single MRI slice, which was shown not to be an influencing factor, and we consider that the BML status at one level alone does not reflect the grade of BML and the impact on prognosis. Although a previous study found that SVF has the potential to reduce bone marrow lesions during short-term treatment [5], we found, in the present study, that BML was not significantly improved at 5 years after SVF treatment compared with the preoperative results and compared with the data in the control group, suggesting that SVF injection in the joint cavity does not seem to improve BML in the subchondral bone. Intra-articular injections supplemented by subchondral injections may be an option to try. The most direct correlation between BML and clinical symptoms is pain, and intolerable pain is often the immediate cause of patients seeking medical treatment. A 6-month retrospective study [44] showed that the pretreatment presence of BML was also associated with daily activities and function in the short term, suggesting that BML is more responsive to PASS regardless of pain or function. Since preoperative cartilage factors are not influential factors in prognosis for clinical failure, we believe that sufficient attention should be given to BML severity in serial MRI slices, as this may imply a higher risk of failure with the treatment of SVF. Therefore, we continue to recommend active intervention with BML.

The KL grade is the most widely used method in clinical practice for assessing KOA severity. A 2-year follow-up of 30 patients undergoing stem cell therapy found that the KOOS score of KL grade 2 was superior to that of grade 3 [45]. Another study showed that the KL3 group improved more than the KL2 group after SVF treatment [5]. We compared the prognosis of KL grades 2 and 3, which turned out not to be an independent risk factor. The KL grade may miss meaningful changes in the bone marrow and cartilage and therefore is not recommended for the evaluation of regenerative therapies [46].

It is well known that obese patients have an increased load on weight-bearing joints and thus an increased risk of KOA [47]. A cross-sectional study showed a dose–response relation between high BMI and pain and function in patients with KOA [48]. This may also apply to prognostic analysis, where patients with high BMI are also at increased risk of clinical failure.

There are limitations in the present study. First, the efficacy of SVF in patients with KL grade 4 is unknown. Second, since we performed a simple intra-articular injection without lesion site localization, the exact destination of SVF cells in the joint is unknown, which limits our further understanding of the mechanism of action of SVF. Targeting SVF injections to specific lesion sites and tracking the localization of SVF cells under MRI is a direction for future research.

## Conclusions

Up to 5 years after autologous SVF treatment, acceptable clinical state was present for approximately 60% of patients with less cartilage volume loss. In addition, the high severity of BML and high BMI increased the risk of clinical failure. Intra-articular injection of SVF does not improve subchondral BML.

## Data Availability

The datasets used and/or analysed during the current study are available from the corresponding author on reasonable request.

## Abbreviations

SVF: Stromal vascular fraction
KOA: Knee osteoarthritis
ADSCs: Adipose derived stem cells
HA: Hyaluronic hcid
VAS: Visual analogue scale
WOMAC: Western Ontario and McMaster University Osteoarthritis Index
PASS: Patient acceptable symptom state
BML: Bone marrow lesion
BMI: Body Mass Index
VIF: Variance inflation factor
KL: Kellgren–Lawrence

## References

[1] Zhang Y, Jordan JM (2010). Epidemiology of osteoarthritis. Clin Geriatr Med.

[2] Jevotovsky DS, Alfonso AR, Einhorn TA (2018). Osteoarthritis and stem cell therapy in humans: a systematic review. Osteoarthr Cartil.

[3] Charles-de-Sá L, Gontijo-de-Amorim NF, Maeda Takiya C (2015). Antiaging treatment of the facial skin by fat graft and adipose-derived stem cells. Plast Reconstr Surg.

[4] Semon JA, Zhang X, Pandey AC (2013). Administration of murine stromal vascular fraction ameliorates chronic experimental autoimmune encephalomyelitis. Stem Cells Transl Med.

[5] Tran TDX, Wu CM, Dubey NK (2019). Time- and Kellgren-Lawrence grade-dependent changes in intra-articularly transplanted stromal vascular fraction in osteoarthritic patients. Cells.

[6] Hong Z, Chen J, Zhang S (2019). Intra-articular injection of autologous adipose-derived stromal vascular fractions for knee osteoarthritis: a double-blind randomized self-controlled trial. Int Orthop.

[7] Garza JR, Campbell RE, Tjoumakaris FP (2020). Clinical efficacy of intra-articular mesenchymal stromal cells for the treatment of knee osteoarthritis: a double-blinded prospective randomized controlled clinical trial. Am J Sports Med.

[8] Ding C, Cicuttini F, Jones G (2007). Tibial subchondral bone size and knee cartilage defects: relevance to knee osteoarthritis. Osteoarthr Cartil.

[9] Everhart JS, Abouljoud MM, Kirven JC (2019). Full-thickness cartilage defects are important independent predictive factors for progression to total knee arthroplasty in older adults with minimal to moderate osteoarthritis: data from the osteoarthritis initiative. J Bone Joint Surg Am.

[10] Yusup A, Kaneko H, Liu L (2015). Bone marrow lesions, subchondral bone cysts and subchondral bone attrition are associated with histological synovitis in patients with end-stage knee osteoarthritis: a cross-sectional study. Osteoarthr Cartil.

[11] Klement MR, Sharkey PF (2019). The significance of osteoarthritis-associated bone marrow lesions in the knee. J Am Acad Orthop Surg.

[12] Nielsen FK, Egund N, Jørgensen A (2017). Risk factors for joint replacement in knee osteoarthritis; a 15-year follow-up study. BMC Musculoskelet Disord.

[13] Zhang Y, Nevitt M, Niu J (2011). Fluctuation of knee pain and changes in bone marrow lesions, effusions, and synovitis on magnetic resonance imaging. Arthritis Rheum.

[14] Hochberg MC, Altman RD, Brandt KD (1995). Guidelines for the medical management of osteoarthritis. Part II. Osteoarthritis of the knee. American College of Rheumatology. Arthritis Rheum.

[15] Kellgren J, Lawrence J (1957). Radiological assessment of osteo-arthrosis. Ann Rheum Dis.

[16] Jones G, Glisson M, Hynes K (2000). Sex and site differences in cartilage development: a possible explanation for variations in knee osteoarthritis in later life. Arthritis Rheum.

[17] Dore D, Quinn S, Ding C (2010). Natural history and clinical significance of MRI-detected bone marrow lesions at the knee: a prospective study in community dwelling older adults. Arthritis Res Ther.

[18] Zhai G, Blizzard L, Srikanth V (2006). Correlates of knee pain in older adults: Tasmanian Older Adult Cohort Study. Arthritis Rheum.

[19] Tubach F, Ravaud P, Baron G (2005). Evaluation of clinically relevant states in patient reported outcomes in knee and hip osteoarthritis: the patient acceptable symptom state. Ann Rheum Dis.

[20] Saag KG (2003). OMERACT 6 brings new perspectives to rheumatology measurement research. J Rheumatol.

[21] Song Y, Du H, Dai C (2018). Human adipose-derived mesenchymal stem cells for osteoarthritis: a pilot study with long-term follow-up and repeated injections. Regen Med.

[22] Ding C, Cicuttini F, Blizzard L (2007). A longitudinal study of the effect of sex and age on rate of change in knee cartilage volume in adults. Rheumatology (Oxford).

[23] Ding C, Cicuttini F, Scott F (2005). Association between age and knee structural change: a cross sectional MRI based study. Ann Rheum Dis.

[24] Guo J, Nguyen A, Banyard DA (2016). Stromal vascular fraction: a regenerative reality? Part 2: mechanisms of regenerative action. J Plast Reconstr Aesthet Surg.

[25] Simunec D, Salari H, Meyer J (2020). Treatment of grade 3 and 4 osteoarthritis with intraoperatively separated adipose tissue-derived stromal vascular fraction: a comparative case series. Cells.

[26] Jacques C, Gosset M, Berenbaum F (2006). The role of IL-1 and IL-1Ra in joint inflammation and cartilage degradation. Vitam Horm.

[27] García JR, Quirós M, Han WM (2019). IFN-γ-tethered hydrogels enhance mesenchymal stem cell-based immunomodulation and promote tissue repair. Biomaterials.

[28] Manferdini C, Maumus M, Gabusi E (2013). Adipose-derived mesenchymal stem cells exert antiinflammatory effects on chondrocytes and synoviocytes from osteoarthritis patients through prostaglandin E2. Arthritis Rheum.

[29] Song WJ, Li Q, Ryu MO (2017). TSG-6 secreted by human adipose tissue-derived mesenchymal stem cells ameliorates DSS-induced colitis by inducing M2 macrophage polarization in mice. Sci Rep.

[30] Ortiz-Virumbrales M, Menta R, Pérez LM (2020). Human adipose mesenchymal stem cells modulate myeloid cells toward an anti-inflammatory and reparative phenotype: role of IL-6 and PGE2. Stem Cell Res Ther.

[31] Morris DL, Oatmen KE, Wang T (2012). CX3CR1 deficiency does not influence trafficking of adipose tissue macrophages in mice with diet-induced obesity. Obesity (Silver Spring).

[32] Dong Z, Peng Z, Chang Q (2013). The survival condition and immunoregulatory function of adipose stromal vascular fraction (SVF) in the early stage of nonvascularized adipose transplantation. PLoS ONE.

[33] Nguyen A, Guo J, Banyard DA, Fadavi D (2016). Stromal vascular fraction: a regenerative reality? Part 1: Current concepts and review of the literature. J Plast Reconstr Aesthet Surg.

[34] Traktuev DO, Merfeld-Clauss S, Li J (2008). A population of multipotent CD34-positive adipose stromal cells share pericyte and mesenchymal surface markers, reside in a periendothelial location, and stabilize endothelial networks. Circ Res.

[35] Zhao X, Ruan J, Tang H (2019). Multi-compositional MRI evaluation of repair cartilage in knee osteoarthritis with treatment of allogeneic human adipose-derived mesenchymal progenitor cells. Stem Cell Res Ther.

[36] Agarwal N, Mak C, Bojanic C (2021). Meta-analysis of adipose tissue derived cell-based therapy for the treatment of knee osteoarthritis. Cells.

[37] Jo CH, Lee YG, Shin WH (2014). Intra-articular injection of mesenchymal stem cells for the treatment of osteoarthritis of the knee: a proof-of-concept clinical trial. Stem Cells.

[38] Pers YM, Rackwitz L, Ferreira R (2016). Adipose mesenchymal stromal cell-based therapy for severe osteoarthritis of the knee: a phase I dose-escalation trial. Stem Cells Transl Med.

[39] Yokota N, Hattori M, Ohtsuru T (2019). Comparative clinical outcomes after intra-articular injection with adipose-derived cultured stem cells or noncultured stromal vascular fraction for the treatment of knee osteoarthritis. Am J Sports Med.

[40] Veronesi F, Berni M, Marchiori G (2021). Evaluation of cartilage biomechanics and knee joint microenvironment after different cell-based treatments in a sheep model of early osteoarthritis. Int Orthop.

[41] Muratovic D, Findlay DM, Cicuttini FM (2018). Bone matrix microdamage and vascular changes characterize bone marrow lesions in the subchondral bone of knee osteoarthritis. Bone.

[42] Roemer FW, Neogi T, Nevitt MC (2010). Subchondral bone marrow lesions are highly associated with, and predict subchondral bone attrition longitudinally: the MOST study. Osteoarthr Cartil.

[43] Radin EL, Rose RM (1986). Role of subchondral bone in the initiation and progression of cartilage damage. Clin Orthop Relat Res.

[44] Higuchi J, Yamagami R, Matsumoto T (2020). Associations of clinical outcomes and MRI findings in intra-articular administration of autologous adipose-derived stem cells for knee osteoarthritis. Regen Ther.

[45] Koh YG, Choi YJ, Kwon SK (2015). Clinical results and second-look arthroscopic findings after treatment with adipose-derived stem cells for knee osteoarthritis. Knee Surg Sports Traumatol Arthrosc.

[46] Cantrell WA, Colak C, Obuchowski NA (2020). Radiographic evaluation of knee osteoarthritis in predicting outcomes after arthroscopic partial meniscectomy. Knee.

[47] Puenpatom RA, Victor TW (2009). Increased prevalence of metabolic syndrome in individuals with osteoarthritis: an analysis of NHANES III data. Postgrad Med.

[48] Raud B, Gay C, Guiguet-Auclair C (2020). Level of obesity is directly associated with the clinical and functional consequences of knee osteoarthritis. Sci Rep.

